# Downregulation of FGF Signaling by *Spry4* Overexpression Leads to Shape Impairment, Enamel Irregularities, and Delayed Signaling Center Formation in the Mouse Molar

**DOI:** 10.1002/jbm4.10205

**Published:** 2019-07-31

**Authors:** Pauline Marangoni, Cyril Charles, Youngwook Ahn, Kerstin Seidel, Andrew Jheon, Bernhard Ganss, Robb Krumlauf, Laurent Viriot, Ophir D Klein

**Affiliations:** ^1^ Program in Craniofacial Biology and Department of Orofacial Sciences University of California San Francisco CA USA; ^2^ Institut de Génomique Fonctionnelle de Lyon Univ Lyon, CNRS UMR 5242, ENS de Lyon, Université Claude Bernard Lyon 1 Lyon France; ^3^ Stowers Institute for Medical Research Kansas City MO USA; ^4^ Faculty of Dentistry, University of Toronto ON Canada; ^5^ Department of Anatomy and Cell Biology Kansas University Medical Center Kansas City KS USA; ^6^ Department of Pediatrics and Institute for Human Genetics University of California San Francisco CA USA

**Keywords:** FGF SIGNALING, ENAMEL MINERALIZATION DEFECT, TOOTH DEVELOPMENT, SPRY4

## Abstract

FGF signaling plays a critical role in tooth development, and mutations in modulators of this pathway produce a number of striking phenotypes. However, many aspects of the role of the FGF pathway in regulating the morphological features and the mineral quality of the dentition remain unknown. Here, we used transgenic mice overexpressing the FGF negative feedback regulator Sprouty4 under the epithelial keratin 14 promoter (K14‐*Spry4*) to achieve downregulation of signaling in the epithelium. This led to highly penetrant defects affecting both cusp morphology and the enamel layer. We characterized the phenotype of erupted molars, identified a developmental delay in K14‐*Spry4* transgenic embryos, and linked this with changes in the tooth developmental sequence. These data further delineate the role of FGF signaling in the development of the dentition and implicate the pathway in the regulation of tooth mineralization. © 2019 The Authors. *JBMR Plus* is published by Wiley Periodicals, Inc. on behalf of American Society for Bone and Mineral Research.

## Introduction

Teeth develop through a series of signaling interactions between dental epithelium and the underlying mesenchyme. Epithelial morphogenesis serves several crucial functions during mammalian tooth development, or odontogenesis, because it drives the shape of the cusps that make up the dental crown. Molar patterning is determined by positioning of successive signaling centers (primary and secondary enamel knots) that form where cusps will be present.^1^ These tightly regulated developmental steps determine species‐specific cusp patterns that cannot be remodeled once molar eruption occurs.

In addition to its function in skeletal development,^2^ FGF signaling is a central regulator of tooth development. The role of Fgf genes in this setting has been investigated using mutants for ligands and receptors,^3^, ^4^, ^5^, ^6^ modulators of the pathway,^7^ and interactors like members of the Bmp pathway.^8^ Research in the field has focused on dissecting the function of the pathway in determining tooth shape^9^, ^10^ and has also shed light on the potential implication of this pathway in the evolution of the complex mammalian molar.^11^, ^12^, ^13^, ^14^

Sprouty (Spry) genes were first identified as inhibitors of signaling through FGF receptors (FGFRs) in tracheal morphogenesis in *Drosophila*, and soon after these findings were extended to the mouse.^15^ Four Sprouty orthologs are found in the *Mus musculus* genome,^16^ and *Spry1*, *Spry2*, and *Spry4* are expressed during tooth development.^7^ Their expression is induced upon growth factor stimulation, and the protein products inhibit FGFR‐mediated activation of the ERK‐MAPK signaling pathway.^17^ In the mouse, *Spry2* and *Spry4* prevent the development of supernumerary teeth,^7^ and *Spry1*, *Spry2*, and *Spry4* are required for correct molar cusp patterning.^18^, ^19^ In the mouse incisor, which is a continuously growing tooth, *Spry2* and *Spry4* restrict the differentiation of enamel‐secreting ameloblasts to the labial side, allowing asymmetric enamel deposition.^20^

Here, to further investigate the roles of the FGF signaling pathway in odontogenesis, we utilized a transgenic mouse line (K14‐*Spry4*) in which the expression of mouse *Spry4* is driven in the epithelium of many ectodermal organs under the control of the human keratin‐14 promoter. This line was designed to attenuate epithelial FGF signaling. Although in the course of tooth development *Spry4* is normally expressed in the dental mesenchyme,^7^ the K14‐*Spry4* transgene is expressed throughout the oral epithelium, including the dental epithelium. The erupted molar morphology in the transgenic specimens displays numerous signs of enamel mineralization defects along with variable cusp defects. Histological analyses of the developing molar germs highlight a developmental delay that affects the formation of the tooth signaling center known as the primary enamel knot (pEK). These findings further establish FGF signaling as a critical regulator of enamel mineralization and confirm its role in controlling tooth shape.

## Material and Methods

### Transgenic mice

K14‐*Spry4* mice have been previously reported.^21^ The line was maintained by breeding hemizygous transgenic males with C57Bl/6 J females. Mice were housed at the Laboratory Animal Resource Center (University of California, San Francisco, CA, USA). The transgenic offspring were readily recognizable by sparse, abnormal fur. Although we expected to get approximately 50% transgenic embryos in each litter, we found a decrease in the transgenic embryo proportion starting at embryonic day (E) 16.5 (Mann‐Whitney Wilcoxon sum rank test, *p* value* <*0.05; Supplemental Table 1).

### Characterization of erupted dentition

Twenty‐five transgenic adults and 15 WT littermates were used. At 5 weeks, animals were euthanized by CO_2_ asphyxia followed by cervical dislocation. Bony heads were cleaned by a colony of *Dermestes maculatus* beetles and radiographed using a Phoenix Nanotom S (GE Measurement and Control, Billerica, MA, USA) with a tungsten source X‐ray tube operating at 100 kV and 70 μA. The Phoenix datosc2CT software was used to compute a reconstruction of the 3D volumes, with a final voxel size of 3 μm. The crown surface was measured on the occlusal‐oriented pictures of the scanned volumes by drawing the outline of the molars. Virtual segmentation of enamel and enamel thickness calculation and mapping were performed using Amira software (version 6.2; Thermo Fisher Scientific, Waltham, MA, USA). Thickness was defined as the distance along the vertex normal to the normal's intersection with the closest enamel surface (external surface or enamel–dentin junction). To avoid the biases caused by worn enamel surfaces, we decided to extract the mode as representative of the enamel thickness of each sample.

### Enamel microstructure analysis

Cleaned upper and lower molar rows were fixed in 4% PFA in PBS overnight, then dehydrated in a graded ethanol series and dried in a vacuum desiccator. After being embedded in epoxy resin (resin 105 and hardener 205 at a ratio of 5:1 w/w; WestSystem, Bay City, MI, USA), they were ground to the desired thickness on a plate grinder (EXAKT 400CS; EXAKT Technologies, Oklahoma City, OK, USA) using 800‐grit silicon carbide paper and polished with 2000‐ and 4000‐grit silicon carbide paper (Hermes Abrasives, Mississauga, ON, Canada). The exposed tissue was etched with 10% phosphoric acid for 30 s, rinsed with water, and dried in a vacuum desiccator. Samples were mounted on SEM stubs with carbon tape, surfaces coated with 7‐nm gold using a sputter coating machine (Desk II; Denton Vacuum, Moorestown, NJ, USA), and imaged in a Philips SEM instrument (XL30 ESEM, Philips, Andover, MA, USA) operating at a beam energy of 20 keV. Images were processed using Adobe Photoshop CS5.1 (Adobe, San Jose, CA) to adjust upper and lower limits of input levels in grayscale mode, and to apply auto balance and auto contrast settings.

### Histological analyses

Noon of the day the vaginal plug was detected in breeding females was considered as E0.5. Entire litters (total of 125 embryos) were collected every 12 hours from E11.5 (about 12 hours postodontogenesis initiation) to E17.5 (after the beginning of first molar mineralization). WT and transgenic littermates were genotyped using the following primers: 5'‐CTGGGCAGGTAAGTATCAAGG‐3' and 5'‐TGGTCAATGGGTAAGATGGTG‐3'. PCR was performed using the following parameters: 2 min at 94°C; 25 cycles of 30 s at 94°C, 30 s at 54.8°C, 1 min at 72°C, and 5 min at 72°C. K14‐*Spry4* transgenic embryos display a 354‐bp fragment specific to the construct.

Embryos were harvested in 1× PBS and fixed overnight in 4% PFA. After dehydration in graded ethanol, embryos were processed in paraffin and serially sectioned (7‐µm thick) using a Leica Autocut 2055 microtome (Leica, Wetzlar, Germany). Masson's trichrome was used to stain the slides generated (hemalum, 8 min; fuchsine, 2 min; aniline blue, 1 min), before samples were imaged using an Olympus microscope (Olympus, Waltham, MA, USA) equipped with a CCD camera and Cell F.

## Results

### Downregulation of FGF signaling leads to enamel irregularities, mild cusp defects, and smaller teeth

**Figure 1:**
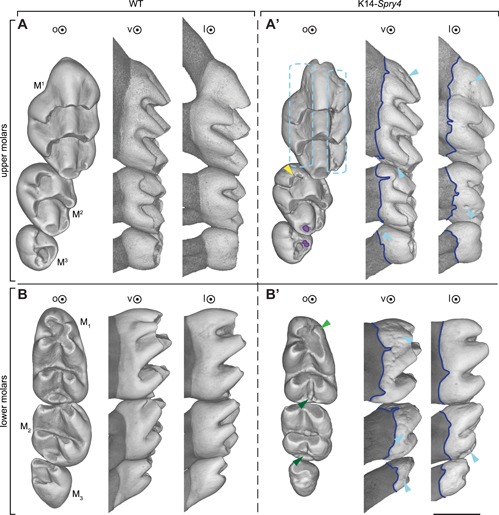
Most prevalent phenotypes in the K14‐*Spry4* mice. (*A*) upper WT molar row, (*A'*) upper transgenic molar row, (*B*) lower WT molar row, (*B'*) lower transgenic molar row. Light blue dotted boxes highlight enamel pitting (62%), navy line shows the irregularities of the enamel–dentin junction (62%), light blue arrowhead points at enamel pearls (20% in upper, 26% in lower molars). Yellow and green arrowheads focus on the main cusp defect in the transgenic molar row: duplication of the M^2^ mesiolingual cusps (yellow, 14%), reduction/absence of the distal‐most cusps of the M_1‐2_ (dark green, 50%), and reduction of the mesiovestibular M_1_ cusp (light green, 30%). Color‐coding matches the description given in Supplemental Fig. 1. o = occlusal view; v = vestibular view; l = lingual view. Scale bar represents 0.75 mm.

We investigated the arrangement and shape of the molar rows in 25 K14‐*Spry4* transgenic mice and 15 of their WT littermates. The mice were collected at 5 weeks of age to study the molar phenotype in fully erupted, but only slightly worn teeth. The molar rows in transgenic mice displayed a variety of defects on both upper and lower molar rows compared with control. In the upper molars, abnormalities were seen in both the mineralized tissues and the cusp pattern. The enamel layer was severely affected by the transgene expression, as evidenced by holes, pitting, and enamel pearls detected on 62% of the specimens and evenly distributed on all tooth faces (Fig. 1 *A*, *A'*; Supplemental Fig. 1). Looking at the occlusal surface, irregularities appeared concentrated along the two mesiodistal valleys of the first upper molar (M^1^; blue boxes, Fig. 1 *A'*). The enamel–dentin junction (between the enamel‐covered crown and the cementum‐covered roots) was irregular on the vestibular, lingual, and/or mesial sides of the molars (44% on the vestibular side only; navy line, Fig. 1 *A'*). Lastly, deep circular dentin wells (diameter approximately 40 µm) were observed on 14% of the molars (purple circle on M^2,3^, Fig. 1 *A'*).

Along with these abnormalities, modifications of the cusp pattern were observed, although to a lesser extent (Supplemental Fig. 2). M^1^ in transgenic mice displayed an ectopic crest linking the lingual cusps of both the first and second chevrons (transversal crests that link the cusps) in 4% of specimens, a disconnection of the lingual‐most cusp of the first chevron (2%), an ectopic crest linking the vestibular cusps of both the first and second chevrons (2%), and a disconnected and individualized first chevron central cusp (2%) (pink through orange; Supplemental Figs. 1, 2). Cusp‐patterning defects were also present on the M^2^, with duplication of the mesiolingual cusp in 14% of samples (yellow, Supplemental Figs. 1, 2).

The enamel appeared irregular in the lower molar rows of the entire cohort, especially on the lingual and vestibular sides of the three molars, with the vestibular side displaying the most severe irregularities (Fig. 1 *B*, *B'*). Irregularities of the enamel–dentin junction were present in the entire transgenic population. The vestibular side was always impacted, whereas 40% of the transgenic cohort also showed irregularities on the enamel–dentin junction on the lingual side (Fig. 1 *B'*, Supplemental Fig. 1).

Moreover, lower M_1_ and M_2_ displayed a more penetrant cusp defect, with the distal‐most part of both teeth reduced or absent in 50% of the transgenic cohort, and the mesiovestibular cusp of the M_1_ reduced in 30% of the transgenic specimens (green arrowheads, Fig. 1 *B'*). Additional cusp defects included an ectopic connection of the distal‐most part of the M_1_ (4%), a bigger mesiolingual cusp (4%), a split mesiolingual cusp (2%), and the presence of cingular cusps (2%; Supplemental Figs. 1, 2).

**Figure 2:**
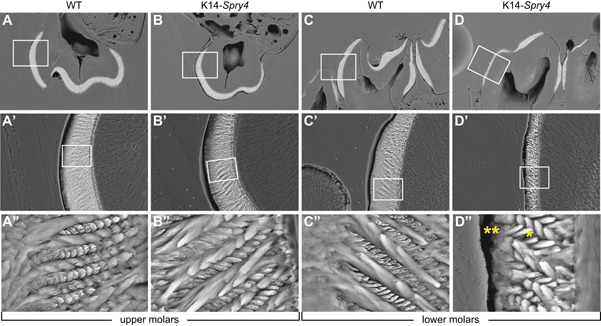
Effects of Fgf downregulation on enamel microstructure in the K14‐*Spry4* mice. (*A–B”*) Etched enamel specimens of second molars from the right hemi maxillas in sagittal view. (*C–D”*) Hemi mandibles in frontal view. A', B', C', D' are zoomed‐in views of the box in *A, B, C, D*, respectively; *A”, B”, C”, D”* are zoomed‐in views of the box in *A', B', C', D'*, respectively. *Indicates a lack of interprismatic enamel. **Indicates poorly developed outer enamel, both in M_2_.

To assess irregularities in the enamel layer further, we conducted a microstructure analysis, which revealed that the enamel in the upper molars appeared indistinguishable in structure between both the WT and transgenic cohorts, as depicted in the magnified views of the vestibular portion of the M^2^ (Fig. 2 *A* to *B”*). However, transgenic enamel was hypoplastic in all mandibular molars (Fig. 2 *C* to *D”*). The magnified views of the distal portion of M_2_ indeed revealed a lack of interprismatic enamel (noted by * in Fig.) and poorly developed outer enamel (noted by ** in Fig.) in transgenic animals compared with WT. Because of this apparent lack of interprismatic enamel in affected molars, the enamel prisms appeared more isolated and clearly demarcated. They also displayed a more compact and less jagged surface topography after etching than the WT enamel prisms.

**Figure 3:**
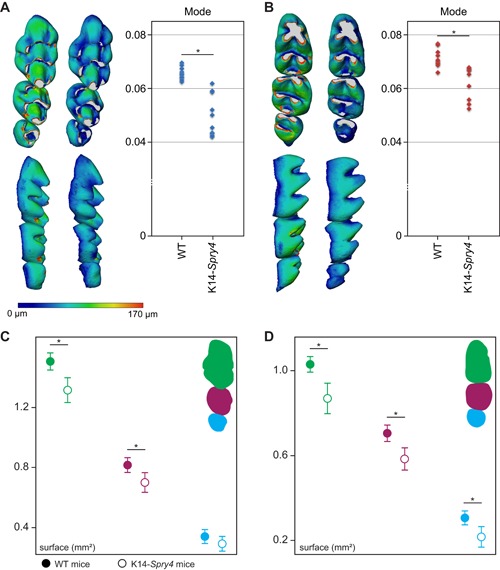
Comparison of enamel thickness and erupted molar surface in the K14‐*Spry4* mice. (*A*) Enamel thickness maps for the upper molars, along with mode quantification. Higher mode reflects a significantly thicker enamel layer in the upper WT molars compared with the transgenic animals (*p* value <0.05). (*B*) Enamel thickness maps for the lower molars, along with mode quantification. Higher mode reflects a significantly thicker enamel layer in the lower WT molars compared with the transgenic animals (*p* value <0.05). The color scale presented in *A* ranges from 0 to 170 μm and is used for both *A* and *B*. (*C*) Measures of the upper molar occlusal surface, M^1,2^ display a significantly smaller surface (*t* test, *p* value <.05). (*D*) Measures of the lower molar occlusal surface, with M_1–3_ displaying a significantly smaller surface (*t* test, *p* value <0.05). WT measures are depicted with filled disks; transgenic measures with blanked ones.

We then sought to quantify enamel thickness, as it appeared from the microstructure analysis that transgenic animals display a thinner layer of enamel. Enamel thickness maps were computed (Fig. 3 *A*, *B*), and the mode for each specimen was extracted as a thickness estimate,^22^ confirming that in both upper and lower transgenic molars, the enamel layer was significantly thinner. Tooth surface measurements confirmed that both the upper and lower transgenic molars were smaller than the WT molars (Fig. 3 *C*, *D*). The measures of tooth surface displayed in the transgenic cohort were more variable than in the WT, consistent with the highly variable additional phenotypes observed in the transgenic mice.

### FGFs are essential to ensure correct pEK formation and proper dental epithelium shape during development

The formation of a group of nondividing cells called the primary enamel knot (pEK) at the cap stage (E14.5 in WT embryos) is necessary for subsequent developmental steps.^23^ The pEK is a cluster of cells that express several growth‐factor encoding genes. These secreted proteins, such as FGF4, direct further invagination of the epithelium, thus playing a role in crown patterning.^24^, ^25^ We first focused on the cap stage, which starts at approximately E14.0 in WT mice (Fig. 3 *C*). Prior to the cap stage, the upper and lower first molar buds from transgenic mice appeared similar to the WT ones (Supplemental Fig. 3). At E14.0, we observed a developmental delay in the transgenic embryos, with the absence of invagination of the cervical loops (black arrowheads in the WT, Fig. 3 *C*). By E14.5, a fully formed pEK was present in all controls, but absent in about 50% of the transgenic embryos (dotted ellipse in the WT, Fig. 3 *C*). Sections also revealed that the developmental delay was less pronounced by E15.5, but the molar germs in the transgenic embryos remained smaller throughout delivery (Supplemental Fig. 3). In addition to the misshapen dental epithelium, a rare but severe fusion of the upper and lower jaws affects 8% of the transgenic cohort, consistent with previous reports.^26^

### *Spry4* overexpression is reminiscent of phenotypes observed in vole molar teeth

**Figure 4:**
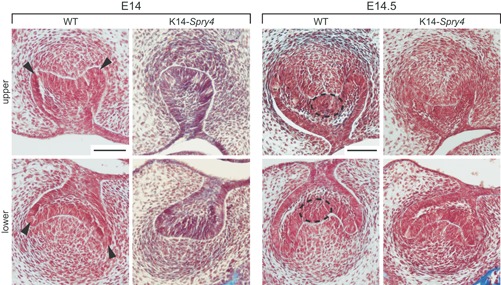
Comparison of cap‐stage molar germ morphology in the K14‐*Spry4* mice. Frontal sections of the upper and lower molar germs in WT and K14‐*Spry4* E14 and E14.5 embryos (*n* = 8 for each time point for transgenic embryos; *n* = 7 and 6, respectively, for WT). Arrowheads point to the delayed invagination of the molar cervical loops (E14), whereas the dotted ellipse shows the absence of a fully formed primary enamel knot by the cap stage (E14.5). Both defects are visible in 50% of the transgenic cohort, and absent in the WT embryos. All sections were stained using Masson's trichrome; scale bars represent 100 µm.

Finally, we compared the transgenic K14‐*Spry4* mice with a specimen of *M. occitanus* from the fossil site of Sète (France), dated at 2.8 Ma^27^ (Fig. 4). Undulations of the enamel–dentin junction are an interesting, but uncommon evolutionary trend during the evolution of mammalian dentition. When this trend is present, it is associated with an increase in tooth crown height (hypsodonty) and allows a better anchorage of the molar through adhesion of dental ligaments in the newly formed enamel‐free areas.^28^ Interestingly, it has been shown that Fgf genes, and especially *Fgf10*, are involved in the transition from low‐ to high‐crowned teeth.^14^, ^29^, ^30^ Although the peaks of the undulated enamel–dentin junction are not positioned exactly at the same locations, this feature might still be the signature of a change in crown‐to‐root transition properties. Our observations thus seem to confirm the pivotal role of the FGF pathway in setting up hypsodonty and related characters during evolution.

**Figure 5:**
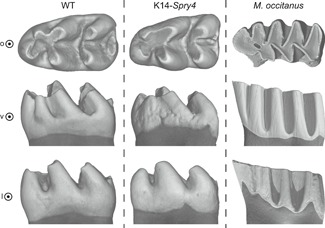
Comparison of the enamel–dentin junction between *Mus musculus* (Murinae) and *Mimomys occitanus* (Arvicolinae). (*A*) *M. musculus*: WT; (*B*) *M. musculus*: K14‐*Spry4*; (*C*) *M. occitanus*: fossil specimen obtained from the UCBL (Lyon, France) collections. o = occlusal view; v = vestibular; l = lingual view.

## Discussion

### The FGF pathway modulates tooth shape

The global downregulation of the FGF signaling pathway in mice carrying the K14‐*Spry4* transgene causes a diminution of the tooth surface. This is similar to what is observed in other Fgf mutants^31^ (namely *Fgf3* ^*–/–*^ mice). The K14‐*Spry4* phenotype includes a variety of discrete shape defects occurring at various frequencies. The phenotypic analysis we have conducted establishes a trend in the reduction of cusp number that highlights the highly refined regulatory network driving molar development, as well as the redundancy between multiple members of the FGF signaling pathway.

The loss of the M_2_ distal‐most cusp mimics the *Fgf3* ^*‐/‐*^ phenotype,^31^ but other abnormalities in K14‐*Spry4* mice have not been described yet in any of the *Fgf* KO mutants. The reduction or absence of the distal‐most parts of both M_1_ and M_2_ also raises the interesting question of the sequence of cusp addition. The molar developmental sequence progresses from the mesial to the distal part of the presumptive row,^11^ but the sequence of cusp formation within a tooth has not been fully characterized yet. Our histological observations during the odontogenic sequence suggest that the delay in forming the pEK truncates odontogenesis with absence of the distal‐most cusps, which would normally be the latest formed.

The display of highly variable cusp defects in both M^1^ and M_1_ might be ascribable to transgene expression variations, resulting in gene dosage changes. It is also interesting to note that the tooth is not the only ectodermal appendage in which development is affected, as these mice also have scarce fur and genitalia defects (data not shown).

### The FGF cascade is a plausible candidate pathway for *amelogenesis imperfecta*

The high frequency of irregularities seen on the enamel layer of the K14‐*Spry4* mice points to the FGF signaling pathway as a regulator of the proper secretion and mineralization of the enamel. The extensive pitting and irregularities observed in both upper and lower transgenic molar rows are reminiscent of human *amelogenesis imperfecta*, a class of autosomal and X‐linked congenital defects occurring with a prevalence of 1:7,000 to 14,000, with pitting, grooves, hypoplasia, defects in color, and softness issues affecting the enamel layer.^32^ Most of the genes implicated in the development of those abnormalities act during the mineralization process.^33^ In the K14‐*Spry4* transgenic line, impaired morphology and thus secretory function of the ameloblasts is linked with global downregulation of FGF signaling. Occasional pits and holes in the dentin and on the root cementum suggest that this role could be extrapolated to other components of the dental matrix. We note that the enamel abnormalities found in our transgenic line differ from those seen in the published *K14‐Cre;Fgfr1* ^*fl/fl*^ mice,^34^ especially in that the impact on enamel appears more severe in the K14‐*Spry4* molars. Together, these findings highlight the potential role of Fgf signaling in the variability seen in *amelogenesis imperfecta* cases clinically.

### Modifications of the dental neck mimic the morphology of certain vole teeth

A major modification of the dental morphology in the K14‐*Spry4* transgenic mice consists in localized rises of the enamel–dentin junction in the three molars of both upper and lower jaws. In the WT embryo, this junction line is largely horizontal and not wavy, but in the transgenic specimens, it is indented in many locations, which results in visible invaginations of the enamel deposition border toward the occlusal surface (Fig. 2). Such a phenotype is reminiscent of the undulations of the enamel–dentin junction observable on the molars of certain fossil voles. In the *Mimomys* lineage (dated from the middle Pliocene,^35^ as depicted with *Mimomys occitanus*, the crown is moderately hypsodont and the undulations of the enamel–dentin junction remain feeble as in K14‐*Spry4* transgenic mice (Fig. 5).

Taken together, our results highlight the importance of FGF signaling in the formation of a smooth and regular enamel layer that covers mouse molars. This signaling pathway regulates the developmental time frame of pEK formation and epithelium morphogenesis. From a clinical point of view, the FGF signaling pathway is a potential candidate that could be modulated to alleviate mineralization defects.

## Disclosures

The authors declare no conflict of interest.

## Supporting information

Supporting information.

Supporting information.

Supporting information.

Supporting information.

Supporting information.
