# Supplementary material for: Downregulation of FGF Signaling by Spry4 Overexpression Leads to Shape Impairment, Enamel Irregularities, and Delayed Signaling Center Formation in the Mouse Molar
Source: JBMR Plus. 2019 Jul 31;3(8):e10205. doi: 10.1002/jbm4.10205 (PMC6715786; doi:10.1002/jbm4.10205)
Supplement: Supplementary file 5 — Supporting information. [file JBM4-3-na-s005.docx]

**Supplemental Table 1: Evolution of both the proportion of transgenic embryos in the collected litters and the mean number of embryos per stage.** The proportion of transgenic embryos drops starting E16.5 (Mann-Whitney Wilcoxon sum rank test, *p-value* < 0.05), while the number of embryos collected in every litter remains consistent (no statistically significant difference detected, *p-value* of 0.1).

**Supplemental Figure 1: Dental character matrix**. (**A,A’**) Upper dental row WT views (**A**) and character matrix (**A’**). The list of characters found abnormal in the K14-*Spry4* specimens is classified based on the tooth concerned (M^1+2+3^, M^1^ or M^2^), colored shapes report the position of the abnormal features, with color coding matching the proportions displayed in the table. (**B,B’**) Lower dental row WT views (**B**) and character matrix (**B’**). The list of characters found abnormal in the K14-*Spry4* specimens is classified based on the tooth concerned (M_1+2+3_, M_1+2_ or M_1_), colored shapes report the position of the abnormal features, with color coding matching the proportions displayed in the table.

**Supplemental Figure 2: Additional cusp defects in the transgenic M^1^ and M_1_.** (**A,B**) Upper and lower WT molar row, respectively. (**A’,B’**) Upper and lower transgenic molar row, respectively. Arrowheads point out at the visible defects, color-coding matches the description given in Supplemental Figure 1. (**A’**) From left to right, transgenic M^1^ also display a connection between the two lingual-most cusps (4%), a disconnection of the lingual cusp of the 1st chevron (2%), a partial vestibular crest (2%), and a splitted and individualized 1^st^ chevron central cusp (2%). (**B’**) From left to right, transgenic M_1_ also display an ectopic connection of the distal-most part of the tooth (4%), a bigger mesio-lingual cusp (4%), a splitted mesio-lingual cusp (2%), and cingular cusps (2%). Scale bar: 0.75 mm. m: mesial; v: vestibular; d: distal; l: lingual.

**Supplemental Figure 3: Dental epithelium displays irregularities and the pEK formation is delayed in the transgenic embryos.** Histo-morphological comparisons of the WT and transgenic M^1^ and M_1_ development. Scale bar: 100 μm, E11.5-E12.5 as in E11.5, E13-E14 as in E13, E14.5-E15.5 as in E14.5, E15-E16 as in E15, E16.5-E17 as in E16.5, E17.5 as indicated.
